# Supplementary material for: Lelliottia amnigena recovered from the lung of a harbour porpoise, and comparative analyses with Lelliottia spp
Source: Access Microbiol. 2023 Nov 9;5(11):000694.v3. doi: 10.1099/acmi.0.000694.v3 (PMC10702373; doi:10.1099/acmi.0.000694.v3)
Supplement: Supplementary material 1 [file acmi-5-694.v3-s001.pdf]

Supplementary figures for Negus *et al.*

Negus D, Foster G, Hoyles L. *Lelliottia amnigena* recovered from the lung of a harbour porpoise, and comparative analyses with *Lelliottia* spp.

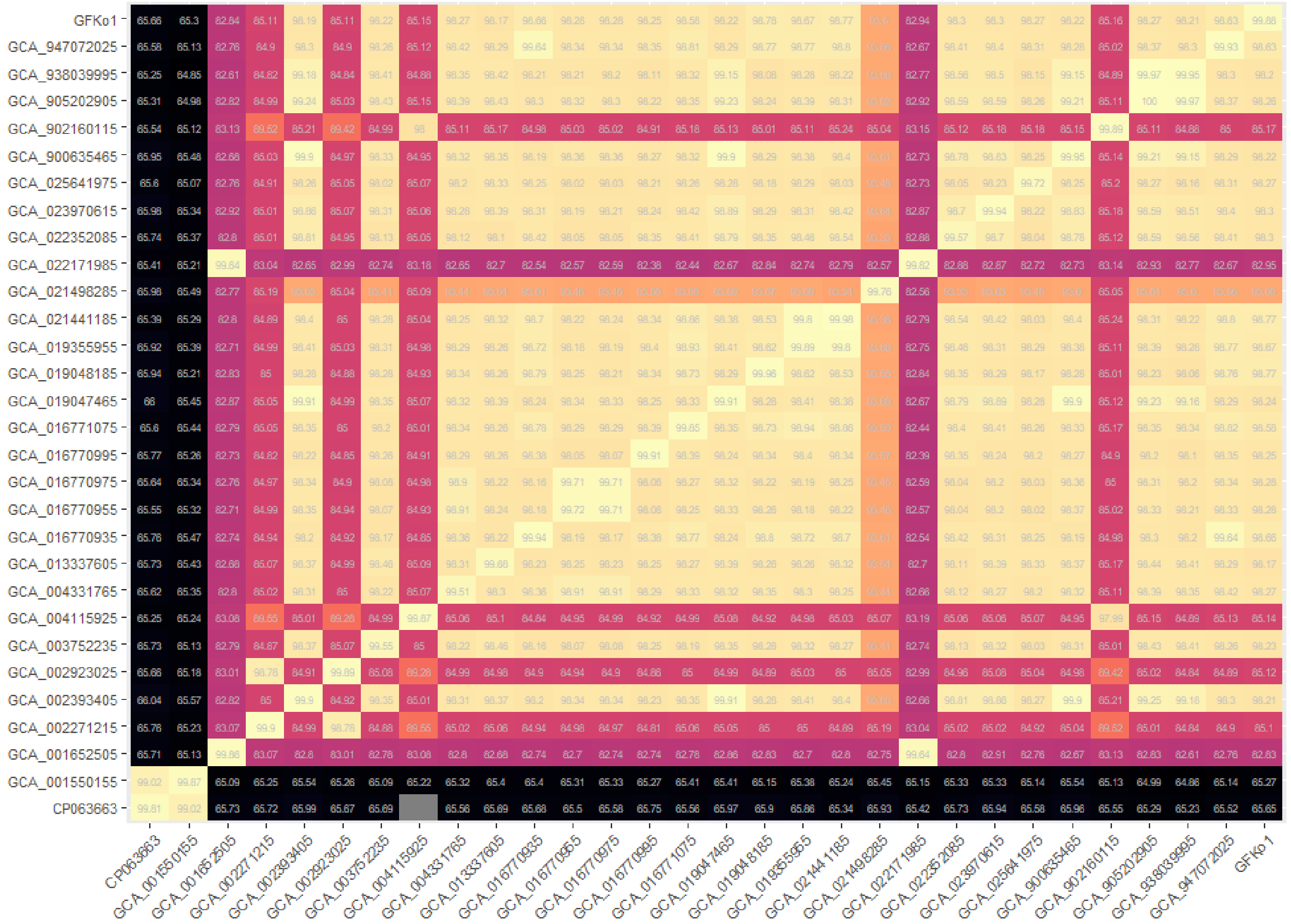

Supplementary Figure 1. Heatmap showing oANI results for all genomes compared with one another. Values in squares represent pairwise oANI (%) values.

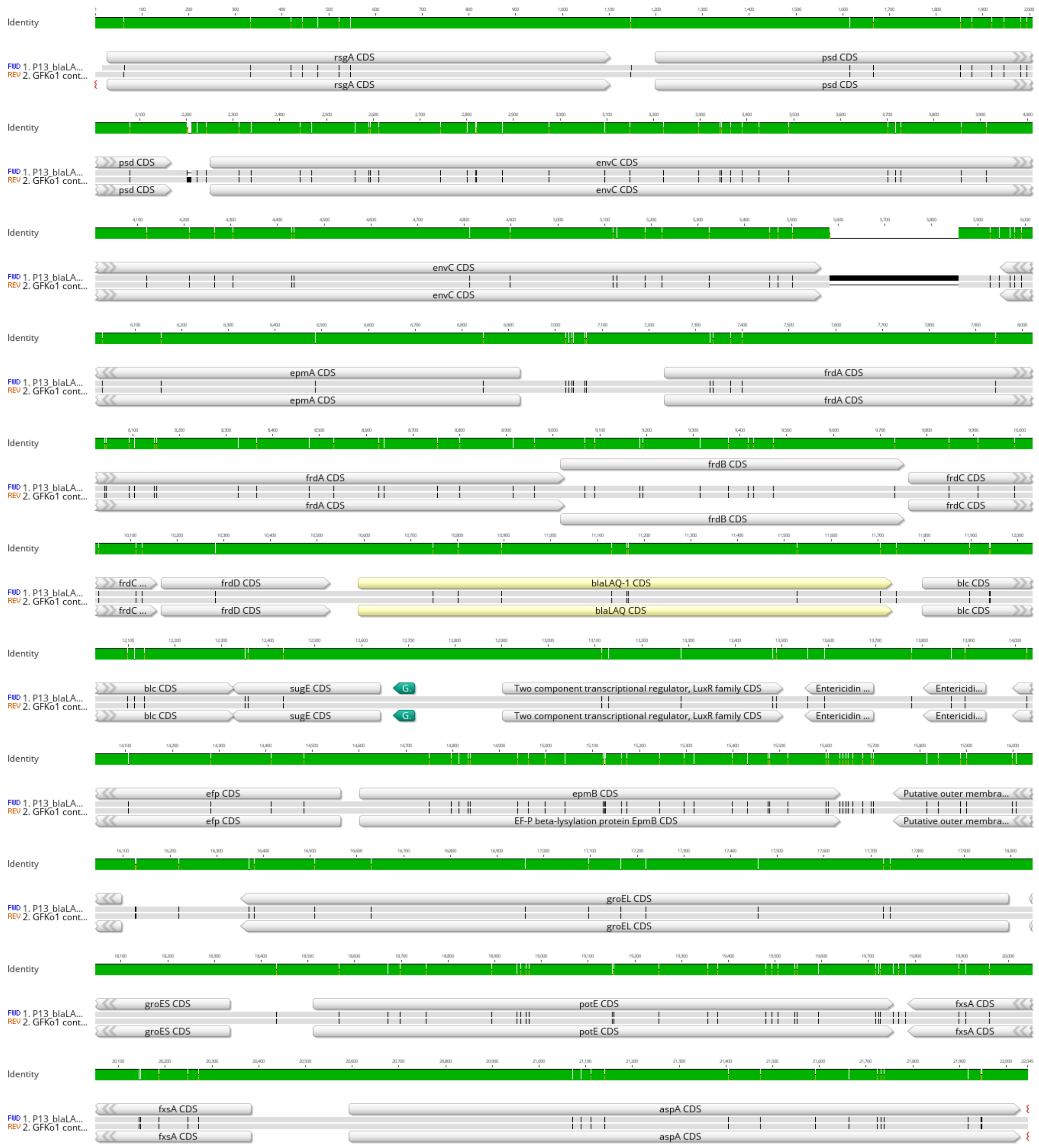

Supplementary Figure 2. Alignment of $\mathit{bla}_{\mathrm{LAQ\text{-}1}}$-like and surrounding genome region of strain GFKo1 with the same region of *L. amnigena* P13. Gene predictions and annotations were made using Bakta as described in Methods. The sequence of GFKo1 was reverse-complemented and the MAFFT alignment shown was created in Geneious Prime v2023.0.1. The alignment covers 21,897 bp; the sequences share 21,497 identical sites (97.6 % pairwise identity) at the nucleotide level. The region shown matches that analysed by (21); their analysis included sequence data from *L. amnigena* strains P13, NCTC 12124^{T}, FDAARGOS 1444, FDAARGOS 1446, FDAARGOS_1445 and FDAARGOS_395.

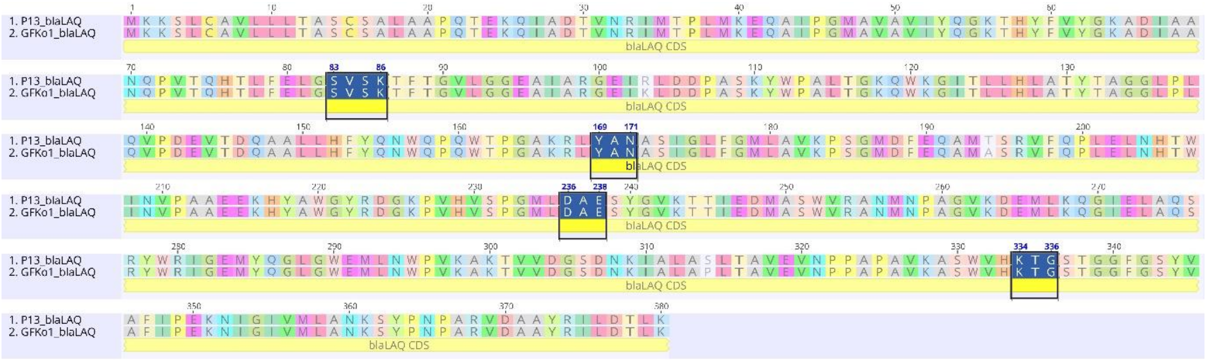

Supplementary Figure 3. Amino acid alignment of β-lactamase LAQ-1 from *L. amnigena* P13 (Accession QXM27670) with β-lactamase LAQ-1 from *L. amnigena* GFKo1 (locus tag GFKo1_06635). Conserved amino acid sequences typical for a class C β-lactamase are highlighted in blue.

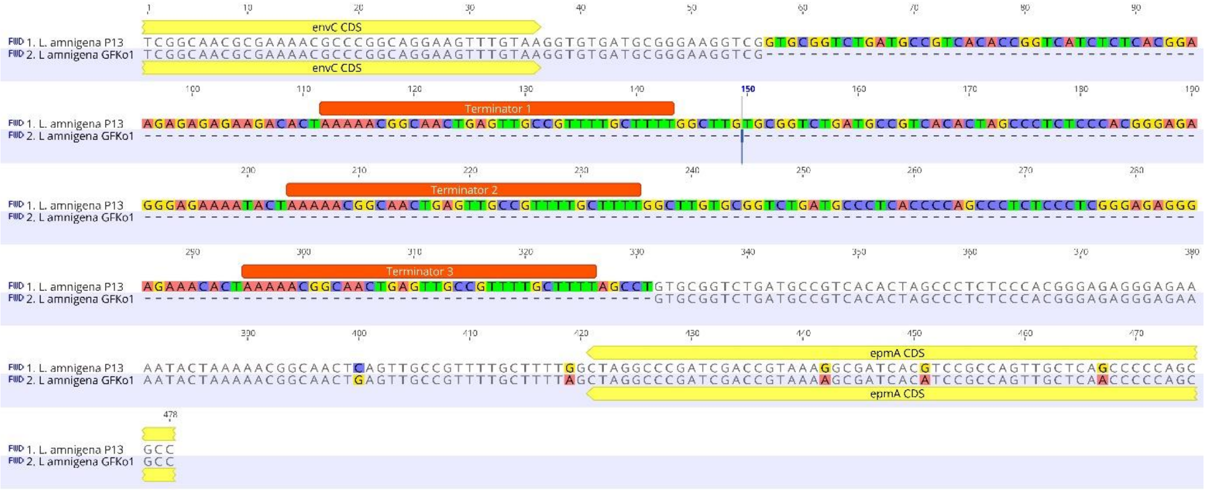

Supplementary Figure 4. Identification of potential transcriptional terminators. Three potential transcriptional terminators were identified in the region between *envC* and *epmA* of *L. amnigena* P13 (orange arrows). This region is absent from *L. amnigena* GFKo1. Terminators were identified using the iTerm-PseKNC tool. Probability scores were >0.99 for all three terminators.
